# Intergovernmental policy opportunities for childhood obesity prevention in Australia: Perspectives from senior officials

**DOI:** 10.1371/journal.pone.0267701

**Published:** 2022-04-28

**Authors:** Emma K. Esdaile, Chris Rissel, Louise A. Baur, Li Ming Wen, James Gillespie

**Affiliations:** 1 Sydney School of Public Health, Faculty of Medicine and Health, The University of Sydney, Sydney, New South Wales, Australia; 2 Charles Perkins Centre, The University of Sydney, Sydney, New South Wales, Australia; 3 NHMRC Centre of Research Excellence for the Early Prevention of Obesity in Childhood, Canberra, Australia; 4 Specialty of Child and Adolescent Health, The University of Sydney, Sydney, Australia; 5 Health Promotion Unit, Population Health Research & Evaluation Hub, Sydney Local Health District, Sydney, Australia; 6 Menzies Centre for Health Policy, The University of Sydney, Sydney, New South Wales, Australia; Drexel University, UNITED STATES

## Abstract

**Background:**

Early childhood (from conception to five years) is a key life stage for interventions to prevent obesity. In the Australian Federation, policy responsibility for obesity prevention sits across all levels of government and several intergovernmental institutions, rendering a complicated policy space. There is a gap in our understanding of the role of intergovernmentalism in developing obesity prevention policy in Australia. Given the complexity of intergovernmental structures and initiatives influencing childhood obesity prevention policy, it is important to understand the perspectives of senior health officials within the bureaucracy of government who through their roles may be able to influence processes or new strategies.

**Methods:**

Document analysis relating to obesity prevention in the intergovernmental context provided material support to the study. This analysis informed the interview guides for nine interviews with ten senior health department officials (one interview per jurisdiction).

**Findings:**

Several opportunities exist to support nutrition and obesity prevention in early childhood including marketing regulation (discretionary choices, breastmilk substitutes, commercial complementary foods and ‘toddler milks’) and supporting the early childhood education and care sector. This study found a widening structural gap to support national obesity policy in Australia. New public management strategies limit the ability of intergovernmental institutions to support coordination within and between governments to address complex issues such as obesity. Subnational informants perceived a gap in national leadership for obesity prevention, while a Commonwealth informant noted the commitment of the national government to partner with industry under a self-regulation model. In this gap, subnational leaders have pursued nationally consistent action to address obesity, including the development of a national obesity strategy as a bipartisan endeavour across jurisdictions. Public officials calculate the strategic possibilities of pursuing opportunities within state agendas but note the limited chances of structural change in the absence of national leadership and funding.

## Introduction

The global increase in the prevalence of childhood obesity over the last few decades [1, 2] has deep systemic causes. It can be seen as a collateral by-product of an integrated global food system and complex transnational patterns of commerce and social change [3]. Addressing childhood obesity requires action spanning governments, industries, environments, communities and families, and public health approaches [4].

Childhood obesity prevention in the early years represents a key opportunity for government intervention for a range of lifelong outcomes [5–7]. There is growing evidence supporting investment in the First 2000 Days (from conception to about five years) as most excess weight in childhood is attained before children start school in Australia [8, 9]. In the 2017–2018 Australian National Health Survey 24.6% of children aged 2–4 years were overweight or obese [10]. Children under five years of age in Australia do not meet core food recommendations [11, 12] and discretionary choices contribute approximately one third of energy intake for children aged 2–3 years [13]. Internationally the First 2000 Days is increasingly recognised as crucial for obesity prevention, however, to date most national childhood obesity prevention policies have focused on school-aged children [14, 15].

Obesity prevention is complex, requiring coordinated policy responses vertically (all levels of government) and horizontally (cross-sectoral) across governments. This paper focuses on the intergovernmental institutions of the Australian Federation relating to obesity policy, and the perspectives of public officials operating in the New Public Management paradigm. It argues for the need for strong intergovernmental mechanisms, funding, and national political leadership to address complex issues like obesity prevention.

### Background: Obesity policy making in the Australian federation

Obesity prevention policies are shaped by political systems. They require “trade-offs between competing interests and values” [16], and considerations of short-term political calculations such as the effects of policy options on public opinion, powerful interests, and prospects for re-election. This ‘political policy lens’ [17] is also affected by deeper, systemic issues and institutional forces–established patterns of power and behaviour which are slow to change. The introduction of policies to confront the obesity crisis provides striking examples of the complex and “explicit engagement with the political and institutional factors affecting the use of health evidence in decision-making” [16].

Australian public policymaking has been shaped by the federal structure of public finance and decision-making. It has also been affected by a high level of political partisanship, especially at the national level. Since the 1980s federal Labor (centre-left) governments have been more sympathetic towards cooperative approaches to federalism, often working with political rivals at state level, whereas Liberal-National (Coalition) (centre-right) governments have been more resistant to the compromises involved in working with the states. Additionally, since the 1980s both sides of politics have been heavily influenced by New Public Management–founded on neoliberalism which concurrently frames our understanding of obesity and forms the governance tools to address it. New Public Management emphasises the efficiency, cost-effectiveness, and productivity of the public sector and public services via results-driven methods of the market system [18–21]. A ‘culture of austerity’ [21] has driven public sector and health service reform across all jurisdictions via New Public Management strategies. These institutional and political structures, and the ideological tensions of New Public Management, have framed intergovernmental will and capacity to prevent early childhood obesity in Australia (see [22–29] for in-depth analysis).

Australian health policy is subject to a pattern of ‘polycentric regulation’ [30]. Decision-making crosses the federal system of Commonwealth national government, six states with constitutionally protected powers and two relatively autonomous territories. The state/territory (subnational) governments have extensive (and costly) responsibilities to deliver health care but have access to few own-source revenues (taxes that they control) and are dependent on fiscal transfers from the Commonwealth government and the conditions that are often attached. This ‘vertical fiscal imbalance’ creates tensions between states’ limited revenue raising capacity and a tendency towards ‘Commonwealth centralisation’ [23]. The Australian Constitution assigns overlapping and contested powers between the levels of government, which can lead to ‘joint decision traps’ (i.e. all levels have to agree or stalemate) or ‘veto points’ (i.e. overlapping power to block change) preventing action on an issue [31]. Such ‘hold out’ powers are referred to as ‘negative coordination’ and can cause gridlock or ‘lowest common denominator outcomes’ [27, 32]. However, confrontation and policy paralysis are less typical than attempts to find paths through the institutional undergrowth.

The Council of Australian Governments (COAG) has been the key forum for multilevel governance in Australia; membership includes Commonwealth, state, territory, and local government representatives. Prior to its establishment in 1992 by a Labor government, Australia had a more ad hoc approach to intergovernmental relations with the central government using its financial powers to direct policy agendas and the states quietly resisting [24]. COAG’s role was to establish greater policy coherence and accountability and smooth out the effects of ‘vertical fiscal imbalance’ and negative coordination [23]. Although agreements can pass with a majority, COAG and its Councils sought consensus. The new approach recognized that policy making was not a zero-sum game. Commonwealth fiscal dominance was matched by state and territory control of implementation. While states can refuse a national agreement, coherent national action was more likely to follow from cooperation rather than coercive use of federal fiscal dominance [27]. States can also join forces to push for policy change, although success of these approaches are rare in the face of Commonwealth fiscal dominance. They require a rare level of agreement across party and regional lines, but are more successful in regulatory policy, where states may control more of the policy instruments.

In 2007, again under a Labor government, the National Preventive Health Agency was established by agreement between states and the Commonwealth, to drive the national agenda in preventive health. The new COAG Reform Council reported on state and territory policy outcomes across a wide array of sectors, including health. These intergovernmental reforms included National Partnership Agreements between the Commonwealth and state and territory governments. The Agreements funded targeted projects rewarding measured outcomes [24]. The National Partnership Agreement on Preventive Health (NPAPH) was Australia’s largest investment in prevention, with food and nutrition policy identified as a central concern [33]. As a National Partnership Agreement, it had significant Commonwealth funding attached allowing governments to invest in the scaling up of programs in many settings. Its Healthy Children Initiative focused on community-based obesity prevention programs. A change to a Coalition government in 2013 saw the termination of the COAG Reform Council along with major Commonwealth-state funding agreements, including the NPAPH. The National Preventive Health Agency was disbanded along with associated governance, reporting, and monitoring infrastructure. The current federal Coalition Government maintains its position that each jurisdiction should be “sovereign in its own sphere” but the loss of the Intergovernmental Agreement on Federal Financial Relations in particular “gave rise to an especially acrimonious period in Commonwealth-state relations” [22].

The abolition of the COAG Reform Council underscored the ‘institutional frailty’ of COAG. Its status remained dependent on the Commonwealth seeing it as a useful policy tool [24]. Successive Coalition governments sidelined COAG due, in part, to its close identification with Labor governments [25, 27]. As a result, so too have national public health policies. Since the end of the NPAPH, Australia has not had a formal overarching national obesity prevention framework. Obesity prevention policy has been shaped–and limited–by these partisan issues and COAG’s institutional structure. While decision-making is shared across governments, considerable power is devolved to industry to self-regulate. The premise of New Public Management has created ideological barriers to public health interventions from both sides of government, where the view is that governments set the general direction of policy but execution is best left to markets and the private sector [18, 21]. This is a tension within Labor, who have been more willing to use policy instruments for intergovernmental cooperation, such as National Partnership Agreements, which accounts for the mixture of policy activism and trust in self-regulation [34].

A recent policy review [14] mapped Australian Commonwealth policies for the early prevention of obesity against the WHO Ending Childhood Obesity Implementation Plan [35]. The review found that Australia had undertaken limited national action in line with the recommendations of the Implementation Plan [14]. These failures were linked to the complexities of Australia’s federal structures and inherent beliefs about who is responsible for childhood obesity prevention. Nationally, ‘personal responsibility’ is the dominant narrative presented for the early prevention of obesity in childhood, shifting attention away from industry regulation to the market preferences of individual consumers [14]. The WHO Ending Childhood Obesity Implementation Plan [35] recommends regulating the marketing of two types of products to prevent obesity across childhood (including early childhood). The Australian responses to these WHO guidelines for these types of products is an industry self-regulation model. The first is the marketing of discretionary choices, supported by the WHO Set of Recommendations on the marketing of foods and non-alcoholic beverages to children. The Commonwealth’s Broadcasting Services Act [36] governs broadcast media but does not set standards for food advertising. These are left to self-regulation by advertising and food industry peak bodies (Australian Association of National Advertisers and Australia Food and Grocery Council respectively). The second is the marketing of breastmilk substitutes (or formula), supported by the WHO International Code of Marketing of Breastmilk Substitutes (WHO Code) and ‘toddler milks’ and commercial complementary foods (for use between 6 to 36 months of age) by subsequent World Health Assembly resolutions (63.14, 63.23, 69.9)) [37]. Australia’s response to the WHO Code is the Marketing in Australia of Infant Formulas (MAIF) Agreement, a self-regulatory voluntary code set by the manufacturers and importers of formula and does not include ‘toddler milks’ or commercial complementary foods. These actions reflect federal political will whose values align with the aims of lobbying by the food and advertising industries, as noted elsewhere in the literature [38–40].

### Background: Obesity prevention and intergovernmental forums

**Fig 1:**
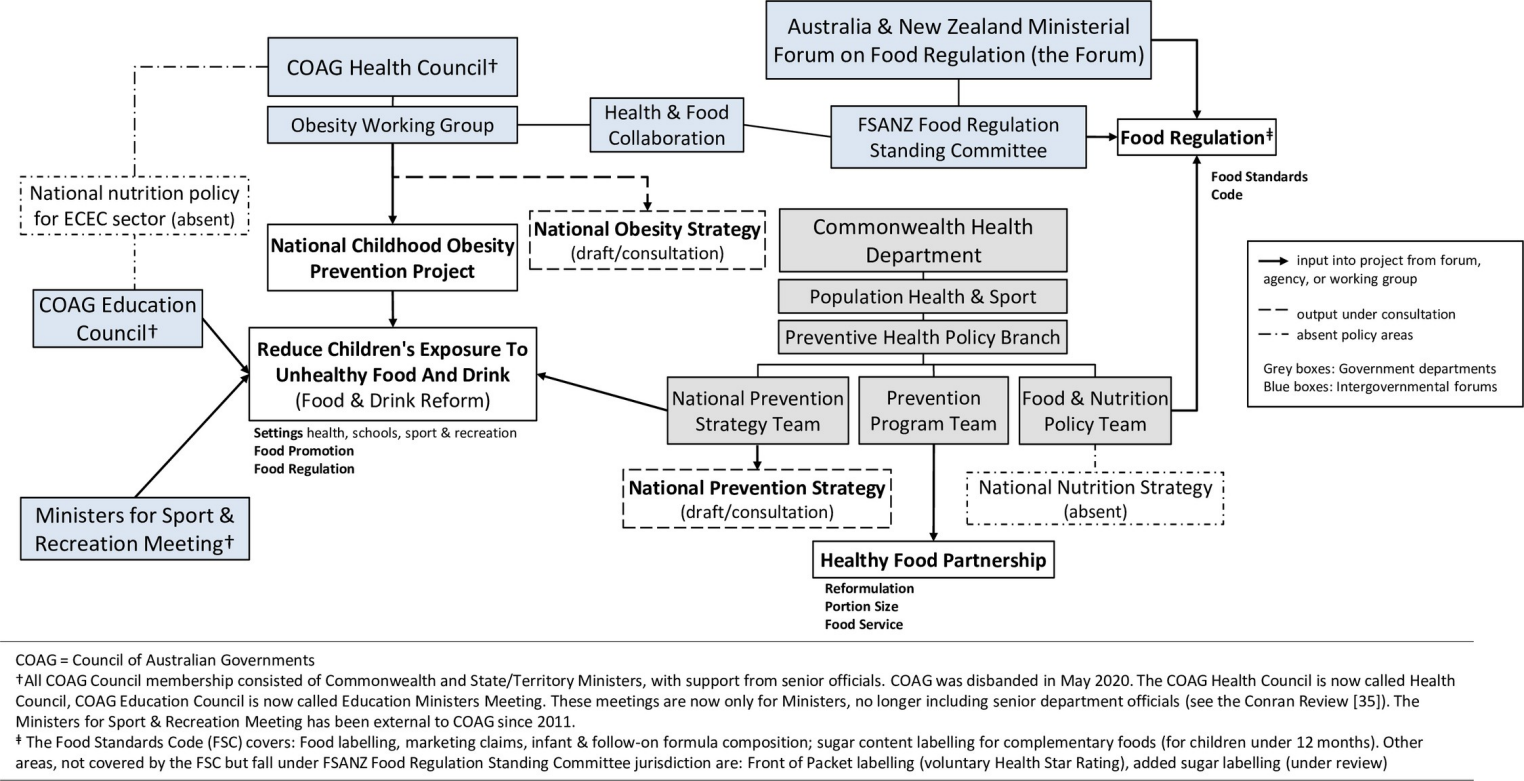
National obesity prevention [42].

To add to the complexity of obesity prevention policy making, health and the Australian food regulation system are jointly regulated by both the national and subnational governments, sitting under two intergovernmental institutions with various councils and subcommittees–COAG and Foods Standards Australian New Zealand (FSANZ). The COAG Health Council is made up of national and subnational health ministers and supported by their respective departments [41]. FSANZ is a bi-national statutory agency with an independent board, including public health and nutrition specialists. FSANZ makes recommendations on food policy through the Australia New Zealand Ministerial Forum for Food Regulation (the Forum), membership includes health and other food-related ministers (e.g. Agriculture) of Australia (national and subnational) and New Zealand. Early iterations of the Forum operated within COAG, but a decision to operate outside of COAG was made in 2013 (see S1 File). Since the removal of the Forum from COAG, the Health and Food Collaboration was established to maintain a connection between the COAG Health Council and the Forum. Beyond these institutions there are other forums where obesity prevention work happens, with several policies in draft through the Commonwealth Health Department–see Fig 1 (for a more in-depth description of these institutions see S1 File).

**Fig 2:**
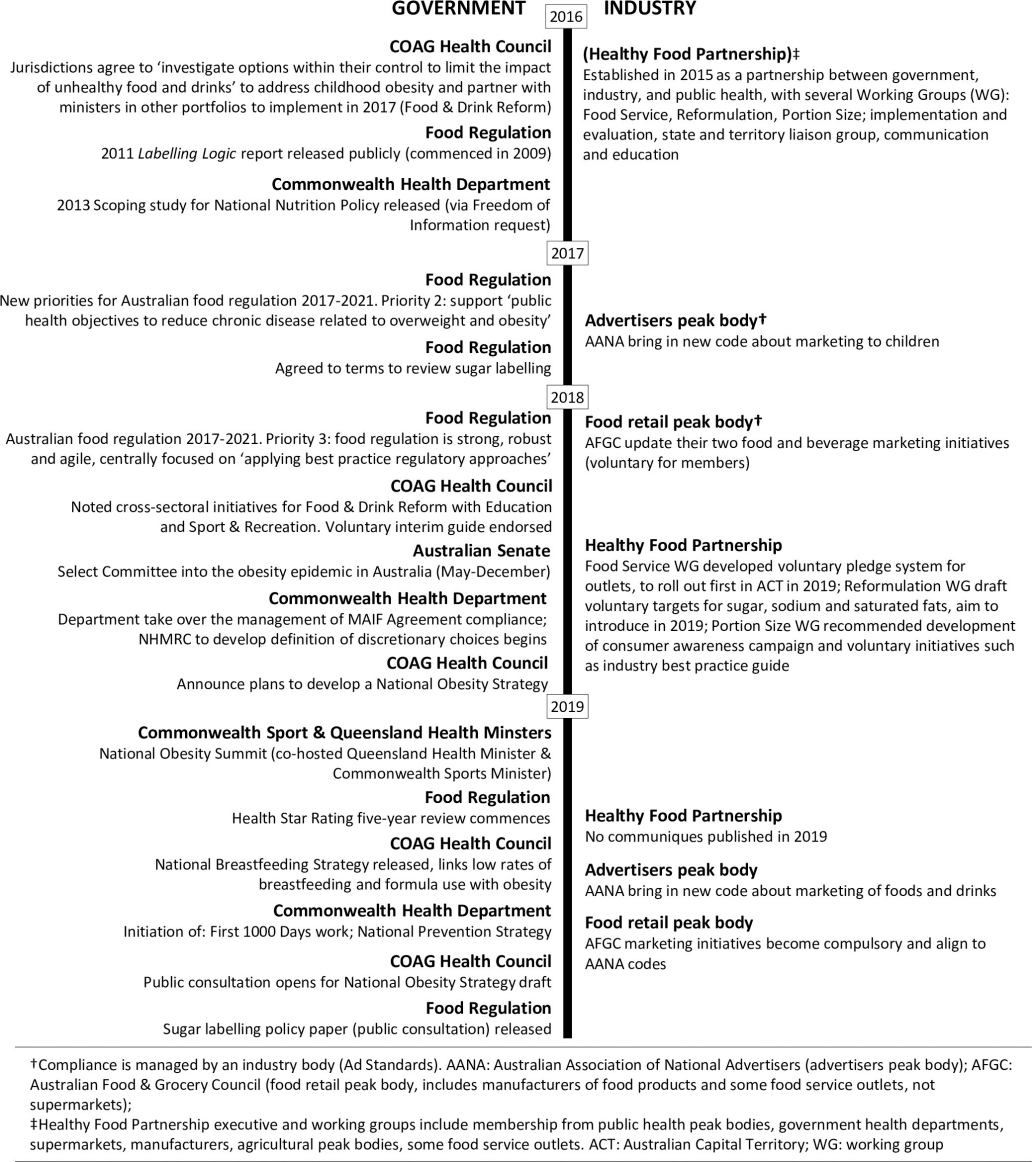
Timeline of recent national obesity prevention activities.

Obesity prevention in Australia is complex, with overlapping responsibilities across jurisdictions, sectors, and institutions such as COAG and FSANZ. In the absence of clear overarching direction to develop a coordinated policy response for obesity prevention, overlapping jurisdictions conflict and block clear lines of policy responsibility. Since the end of the NPAPH there has not been a nationally coordinated approach to obesity prevention. Several pieces of obesity prevention policy have been progressing in intergovernmental forums and through engagement with industry, starting with the National Childhood Obesity Prevention Project in 2016, led by the Obesity Working Group under the COAG Health Council. A summary of activities between 2016–2019 is presented as a timeline in Fig 2.

The *Reduce Children’s Exposure to Unhealthy Food and Drink* project (2016) (which we refer to as Food & Drink Reform in this study) was initiated by the states and driven by the COAG Health Council, not through FSANZ processes. The Food & Drink Reform project had five programs of work. The first three were in settings children were likely to attend (health, school, and sport and recreation spaces), the fourth focused on food promotion within state/territory government control, and the fifth focused on identifying activities within the food regulation system.

In 2018 the Senate Select Committee into the Obesity Epidemic in Australia (Senate Inquiry) was chaired by the Australian Greens Party (a minor left party). In response to Commonwealth government inaction to the Senate Inquiry recommendations, the states announced via the COAG Health Council that they would lead the National Obesity Strategy (for more detail on these pieces of work, see S1 File).

### Study aims

Given the complex intergovernmental structures and initiatives influencing childhood obesity prevention policy, it is important to understand the perspectives of senior health officials within the bureaucracy of government who through their roles may be able to influence processes or new strategies. The aim of this study was to explore the perspectives of senior Commonwealth, state, and territory health department officials about the intergovernmental opportunities for obesity prevention in early childhood. For brevity, this paper focused on a key determinant of obesity, food and nutrition. To the best of our knowledge, there has been no published research on the role of intergovernmentalism in the early prevention of childhood obesity in Australia.

## Methods

### Design

We undertook a qualitative case study of the experiences of senior health officials (in each jurisdiction) relating to the early prevention of childhood obesity in the context of Australian intergovernmentalism. Using semi-structured interviews to provide the content, the data from these interviews were supported by a review of documents to identify initiatives and gaps relating to obesity prevention in Australian intergovernmental forums. Document analysis informed the interview guides and provided context for data interpretation. Our study investigated the current institutional shaping of obesity prevention policies in Australia’s system of multi-level governance. The timeframe under consideration (April 2016 to December 2019) marked new intergovernmental efforts to address childhood obesity since the end of the NPAPH–starting from the COAG Health Council announcement to address childhood obesity through limiting the impact of unhealthy food and drinks to children.

### Participant selection and recruitment

A purposive snowballing sampling strategy was undertaken through the authors professional networks, and those of our colleagues, to identify appropriate respondents in each jurisdictions’ health departments. Potential participants identified were current senior officials within the population health or prevention branches of health departments with responsibility for developing/implementing obesity prevention initiatives in their jurisdiction. Invitations to participate in our study were sent to senior health officers with responsibility for obesity policy in Australian Commonwealth, state and territory health departments. Each potential participant was invited to participate via email containing the study information sheet and consent form and contacted a maximum of three times before the next potential participant was pursued. For more information about study replicability and the transparency criteria used [43] including documentation of interactions and management of power imbalance with participants, see S2 File. In four jurisdictions the initial person contacted agreed to participate. In four other jurisdictions the first person contacted referred our invitation to colleagues within the same branch who they considered more appropriate. Due to internal department restructuring, one jurisdiction required a second round of engagement. In two instances, participants from the same branch chose to be interviewed together, however, this only occurred in one jurisdiction. To support study aims, the minimum requirement for recruitment was at least one participant from each jurisdiction. Interview transcripts were reviewed after each interview to perform first-order coding until at least one interview per jurisdiction had been undertaken and saturation point achieved. This decision was undertaken to ensure the case study had equal representation among jurisdictions.

### Data collection and analysis

To provide material support for the interviews across all Australian jurisdictions, a search was undertaken to identify relevant publicly available documents relating to early childhood obesity prevention. Study authors identified appropriate health-related intergovernmental institutions and Commonwealth/national websites to search for policies and documents relating to early childhood obesity prevention in the context of intergovernmentalism. Periodic reports from intergovernmental institutions were reviewed. For the national websites, key words (child, obesity, healthy, food, physical activity, national) were used in embedded search bars in addition to sitemap reviews. Supportive information for study reproducibility is detailed in S1 File (Part B). The review of documents was undertaken to identify initiatives and gaps relating to national obesity prevention with an early childhood lens, support the development of the interview guides and interpret interview findings.

A semi-structured interview tool was developed based on the study design and the initiatives and gaps identified in the document review (S1 File, Part B). It included informants’ reflections of the NPAPH, their thoughts on what should be considered in the upcoming national obesity strategy, and the intergovernmental institutional mechanisms for communication and collaboration on obesity prevention. Interviews were used to collect data from representatives from national and subnational health departments. In total there were 10 informants including two from the Northern Territory (conducted as one interview). State and territory informants were interviewed by telephone between November and December 2018; the Commonwealth interview was conducted in July 2019. The nine interviews were 61 minutes on average (range: 46–95 minutes). All participants were interviewed by one interviewer. EE is an Accredited Practising Dietitian undertaking this study as part of her PhD candidature. She has professional experience with interviewing and has completed tertiary level training on undertaking qualitative research, including interviewing skills. All interviews were recorded and transcribed verbatim. Informants were given the opportunity to review the transcribed interviews prior to analysis. Interviews were analysed using the Framework Method in NVivo 11 Software.

The Framework Method was developed to manage and analyse data in qualitative research and is commonly used in policy research [44]. A framework was developed for the first-order coding of study data using a range of contextual factors–such as the political nature of childhood obesity, features of the Australian political system, and institutional mechanisms for multilevel governance in Australia [17]–for comparative analysis across a range of institutional settings with obesity prevention in their remit (S1 File). The Framework Method is particularly suited for comparative thematic analysis as it allows researchers to move beyond a description of content from any one participant towards developing themes and offering explanations across the data set [45]. Through this method second-order coding was undertaken in the form of pattern coding [46] to classify the material, determine data themes and develop the study narrative.

Coding was led by EE with dual coding undertaken by LMW and CR to ensure consistency in interpretation. The University of Sydney Human Research Ethics Committee granted ethics approval for this project (Project 2017/507).

## Results

The results are presented in two sections. The first section centres on a key theme that emerged from the interviews with state/territory participants–there was a dominant perception of a lack of national leadership in the obesity prevention space since the end of the NPAPH. The second section centres on the subnational use of intergovernmental mechanisms to pursue national obesity prevention policy formation. Direct quotes from informants are indicated with a ‘P’ followed by a number and jurisdictional indicator (s- state, t-territory, or c-commonwealth), for example: (P1s).

### Lack of national leadership since the NPAPH

#### NPAPH provided good structural and financial support

Study participants were asked to reflect on the NPAPH in the context of the upcoming National Obesity Strategy development. They noted institutional mechanisms–scope and purpose, procedure, designated responsibility/authority, interrelation and cooperation, and funding–through which policies can support obesity prevention. Despite its political demise, the NPAPH had a strong institutional capacity based on a national taskforce and clear governance. Intergovernmental forums set its agendas and directed activities, collaboration, and action among senior policy officers. As a National Partnership Agreement, jurisdictions who signed up to the agreement received financial incentives to reach milestones and this drove “sharing of information and resources” (P2s) to monitor activities and coordinate programs “to try to have some consistency around or agree where we would just be happy to vary different things” (P1s). The standardisation of processes within the Healthy Children Initiative “drove a lot of ideas sharing and collaboration among the states and territories” (P8s).

While the monitoring and reporting structures in the NPAPH were considered useful, the set-up costs were prohibitive in smaller jurisdictions, and several noted they lacked the capacity to obtain timely data for policy decisions:

“You’ve got some jurisdictions where they do a survey every 2 years or something. We just don’t have that capacity to do it” (P7t)

The fiscal imbalance between the Commonwealth and the states/territories created several barriers to program delivery in the states. There were long delays between NPAPH signage (2008) and the money starting to flow out to jurisdictions to implement programs under the Healthy Children Initiative (2011):

“The process by which the money flowed out to the states was quite fraught and bureaucratic, so it did mean there were big delays between the agreements being signed off and money coming to states” (P2s)

While the NPAPH enabled a “boost in investments in prevention” (P2s), soon after there was a change in government (from Labor to a more conservative Coalition) in 2013 the agreement was cancelled. Although some jurisdictions were able to fund Healthy Children Initiative programs to planned completion, few continued after. Over a short period, states experienced an “expansion and then a contraction of investment and activity” (P2s), which had structural and human capital impacts throughout the jurisdictions:

“Nonetheless, when that money was cut, we obviously went through a massive restructure and lost positions and all the rest of it. That then takes time to then get that momentum back again. Because you sort of damage people in that process, even if they’re the ones that survive, they’ll still feel damaged” (P6s)

The NPAPH was seen as a positive initiative that led to useful coordination across Australian jurisdictions, which was ended by the Coalition Government for unknown, but probably ideological, reasons [47].

#### A national plan for nutrition

During the interviews a series of perspectives emerged about specific areas of Australia’s food system. These included a national food and nutrition plan, food and marketing regulation, and fiscal options relating to obesity prevention. Australia does not have an overarching national food and nutrition plan. Study participants noted that states have been anticipating such a plan for “about 25 years” (P9t), and that the considerable work undertaken within the last ten years “…came to nothing” (P2s). Study participants saw the role of a national food and nutrition policy as supporting the population to eat in accordance with dietary guidelines. State and territory informants believed such a plan should be broad and “cover ‘paddock to plate’” (P5s). However, a Commonwealth informant argued nutrition could be adequately incorporated into a National Obesity Strategy and therefore not require a standalone plan:

“What would be the added benefit of a national nutrition policy when we can sort of address it in these other areas?” (P3c)

While this approach reflected the expectations of the Commonwealth government from the public sector, there was concern among subnational study participants that such an approach would be too limited in its scope, as an overarching nutrition policy would be greater than the sum of its parts. Several jurisdictions identified tension between the prevailing view of food as a commodity and public health goals for a food system orientated towards health:

“I would be concerned that if it [nutrition] was only framed under obesity prevention that some of those other aspects might be lost” (P5s)

“[There is a] fundamental problem with our food supply… we actually need a food supply to feed the population in a healthy way” (P6s)

“I think there are some challenges with agriculture’s view of food as a commodity, and engaging with that sector” (P2s)

“I’ve noticed that the call for a national food and nutrition policy is also coming from the agricultural sector and the manufacturing sector” (P7t)

“It’s just a matter of how do we shape what we eat and how do we keep a competitive market domestically, a competitive market internationally in all of these areas. I think it’s just slightly more complex because there are divergent views in that sector” (P3c)

Study participants voiced concerns that the Commonwealth was not taking a leadership role in the nutrition policy space or the obesity prevention policy space:

“We really support the idea of a national obesity strategy… though there needs to be more of a focus on nutrition, less on physical activity. I think physical activity is far less contested in the political sense and is also less of a contributor to overweight and obesity. The energy imbalance elements are far more concerning–not to say that physical inactivity isn’t important, but I think it’s less of a priority at the moment… we would like to see some national leadership on some of those nutrition spaces” (P4t)

Subnational participants noted barriers to developing multi-strategy initiatives were shaped by ideological drivers, often pursuing cost-effectiveness arguments to rationalise investment in prevention:

“…at the moment we go through these incredibly long, drawn-out policy debates around issues. Then they go through long, drawn-out standard development processes in the food regulatory space… It all comes from the political driver, which is around reducing regulatory impact and minimal effective regulation… but the whole driver of minimum effective regulation should be overridden… [because] when it starts driving a food supply in an unhealthy direction, then there’s going to be other costs that come up later” (P6s)

“…a lot of it depends on the political priorities… and it’s also presenting the financial argument around: if you do commit to this, you’re going to be saving a bunch of money down the track” (P7t)

“There’s a lot of rhetoric… particularly at the national level about how important prevention is, but it continues to not get anything like the level of funding… [for] frontline services… As a public servant it has meant having to argue and compete against other government priorities, which is very challenging. I think everyone can see that prevention, it’s a bit of a long game… It’s also quite difficult to make some of the economic arguments around it because attribution is so challenging” (P4t)

In the absence of an overarching national obesity prevention framework since the end of the NPAPH, or a national food/nutrition plan, states and territories have used intergovernmental forums to progress national obesity prevention activities.

#### Commonwealth-only policy areas

A recurring theme identified by subnational study informants was the absence of national leadership in both shared and Commonwealth-only policy areas. Commonwealth-only policy areas include regulatory options such as marketing of discretionary choices on broadcast and digital/online media, marketing of breast milk substitutes and fiscal options such as a health levy or tax on sugar-sweetened beverages.

Marketing of discretionary choices to children was considered “critical” (P9t) among study participants for childhood obesity prevention. The Commonwealth Health Department does not have the authority to regulate broadcast and digital/online media advertising:

“We would like to see those strengthened [but]… it’s not necessarily us that can strengthen them… we need to partner with others to help them determine what the criteria are for what could/should be advertised to children. And then working with the industry to initially strengthen their own voluntary codes. That’s the way this government is operating to really work with others to enhance what they’re already doing rather than coming in and regulating in the area” (P3c)

Just as the Commonwealth Health Department does not have the authority to regulate marketing of discretionary choices, the Department also does not have the authority to regulate the marketing of formula, complementary foods, and foods/drinks marketed to young children. In 2013, “responsibility for adherence to MAIF [the Marketing in Australia of Infant Formulas Agreement]” (P3c) was moved from the Commonwealth Health Department to industry self-monitoring. In 2017 this role was taken back by the Department, implying industry self-monitoring was insufficient to ensure the MAIF Agreement was honoured. Even so, the Department has little recourse to initiate punitive measures for violations of adherence to the MAIF Agreement. A Commonwealth Health Department participant noted the department “would like to see a review of” (P3c) the MAIF Agreement, a position supported by other participants who felt that “MAIF is a little bit too weak” (P7t). Areas the Commonwealth Health Department were considering at the time of data collection included a review of how infant and follow-on formulas (includes birth to 12 months) were covered, and in addition ‘toddler milks’ (from 12 months) which–like the Foods Standards Code–were not covered by MAIF:

“My team has initiated some initial work in this area… So that’s the sort of bucket we are considering within the MAIF discussion [infant formula, follow-on formula, and toddler milks] are all products that sit on the same shelf and are marketed reasonably in the same way” (P3c)

While complementary foods are a component of the WHO Code and subsequent World Health Assembly Resolutions, they were not under consideration in the ‘MAIF discussion’. Furthermore, comments from a study participant reinforced the unlikelihood of pursuing regulatory approaches under the Coalition government:

“So again, it really is about partnership in this area and actually working out what our expectation is of our adherence to the WHO [Code], but also what public health expects and what industry expect and actually working through that to come up with what we would consider a reasonable outcome of that… I wouldn’t suggest that we’re looking to regulate in this area just now” (P3c)

In addition to limitations of national political will to regulate, neither the Government nor the Opposition support fiscal interventions [48]. Fiscal policy options as levers for obesity prevention, such as a sugar-sweetened beverage levy, are beyond health and food regulatory systems as fiscal policy sits with the Prime Minister and Cabinet, including Treasury. In the current political environment study participants recognised significant barriers to implementing such a policy:

“When you’ve got industry on board who are influencing policy, I think that’ll be a big challenge to get something like that through, a sugar tax while they’ve got so much power at the table. Just thinking clearly about who gets a say and who is invited. Things like that get derailed” (P9t)

“WA Health Minister has come out and said that in Western Australia that they want the Commonwealth to introduce the sugar tax” (P1s)

“We know that addressing the consumption of sugar sweetened beverages is a priority across a range of child and adult settings, so a sugar tax is aspirational, but is certainly something that would have an impact” (P5s)

However, the interviews with the Commonwealth informant made it clear that regulatory and fiscal measures were not a consideration, and that regulation would only be a consideration where a ‘market failure’ could be shown,

“…the Liberal-National [Coalition] government is more committed to working with others rather than to regulate… It’s only when you’d see a market failure that you might step in and regulate that area… If you had something in place and everyone was meeting it but people didn’t think it was strong enough, that’s not necessarily a market failure. The market failure comes if you had something in place and the industry weren’t meeting it that would be the market failure in this particular area. So I think yes, there are levers in place that we can pull if there are failures in these areas” (P3c)

In the absence of Commonwealth leadership, subnational leaders have pursued a new national strategy for obesity prevention.

### Subnational use of intergovernmental mechanisms

This section focuses on activities through the COAG Health Council and FSANZ/the Forum which progressed in the absence of national obesity strategy and national food/nutrition plans.

#### National Childhood Obesity Prevention Project

The COAG Health Council’s National Childhood Obesity Prevention Project evolved over time. The project focused on a key upstream commercial area for obesity prevention (which influences child health in general), reducing children’s exposure to marketing of unhealthy foods and beverages:

“the COAG preventing child obesity… It’s not actually child obesity, it’s unhealthy food and drink marketing to children” (P1s)

The Food & Drink Reform project sought coordination in two ways: collaboration across sectors and policy harmonisation across jurisdictions, seeking “some national coordination of things” (P1s) within subnational policy control. Largely the first three Food & Drink Reform programs affected school aged children rather than early childhood, as early childhood education and care (ECEC) settings were not included. Current interventions to support children during the early years, in the ECEC sector, to achieve appropriate nutrition and physical activity in line with the national regulatory framework are primarily programs driven by state and territory health departments and are absent from COAG Education Council communiques.

The Australian Capital Territory decided to remove advertising of discretionary choices from their trains and buses as a strategy to address childhood obesity. An informant noted “quite a lot of interest” (P4t) in the policy among jurisdictions at the Food & Drink Reform meetings, relating to its fourth program. A notional link can be drawn from there to the Queensland government’s announcement to apply a similar policy to all government-owned outdoor advertising sites (excluding stadia). The Western Australian government has removed alcohol advertising on public transport assets, to support their public health aim of reducing alcohol consumption and alcohol-related harm. Extending this policy to cover discretionary choices and their childhood obesity prevention aim will be considered in the future:

“[That] has been raised… I would expect the government probably wants to see how that [alcohol advertising removal] plays out before it looks to expanding that to junk food” (P2s)

South Australia has developed good will internally with a whole-of-government approach to health policy (Health-in-All-Policies, which commenced under a Labor government and continued under a Coalition government) and are exploring ways to apply the fourth Food & Drink Reform program:

“We’re in the process of using our Health in All Policies processes to link in with the decision-making processes of the new government to try to actually get whole of government buy-in to do this”

However, restrictions on discretionary choices marketing on government assets have not been implemented in South Australia to date. Despite Food & Drink Reform’s fourth program also having the potential to be applied to Commonwealth-controlled settings, that was not something being actively pursued, “we at the Commonwealth haven’t put some thought into that yet” (P3c).

Under the Food & Drink Reform project’s fifth program, the food regulation system was engaged to “achieve outcomes” (P3c) of the Food & Drink Reform project. This engagement linked intergovernmental forums and contributed to food regulation system priority changes in 2017, including supporting efforts to prevent chronic disease [49]. Two pieces of work were being considered under this program: the definition of discretionary choices and menu labelling. Menu labelling was initially implemented in New South Wales in 2011, followed by other jurisdictions each of which implemented the policy in different ways. Since many food outlet businesses in Australia exist in more than one jurisdiction, participants noted that the different applications of menu labelling policy were confusing for industry:

“I honestly have huge sympathy for industry, because they’ve got different guidelines to cope with in different jurisdictions” (P6s)

An outcome of this program was guidance on ensuring a nationally consistent approach to menu planning legislation for all jurisdictions, should they choose to implement it.

The term ‘discretionary choices’ was defined in the Australian Dietary Guidelines in 2013 [50], however, a Commonwealth participant noted that there was confusion “about what a discretionary food is” (P3c). Since national guidelines are supposed to be reviewed every five years the Food & Drink Reform work presented an opportunity to review the term. The Commonwealth Health Department commissioned the National Health and Medical Research Council (NHMRC) to look at how ‘discretionary choices’ was being used by different organisations including industry, government and public health:

“And that piece of work did show that we [government and industry] are using it differently in different settings. So, there is a need for a better definition for these products…But it’s still got a little way to go” (P3c)

The NHMRC Discretionary Foods and Drinks Expert Working Group (five members from industry, media, not for profit sector, public health, and academia) were due to report to the NHMRC CEO in June 2020, but a decision was taken to delay the definition review until a full review of the Australian Dietary Guidelines could be undertaken.

While the Food & Drink Reform was dedicated to developing nationally consistent approaches in this space, this work was voluntary and had no funding attached. Study participants reflected that its implementation is likely to be sporadic:

“I have seen a couple of jurisdictions leading, which is great, and we hope that many will follow. It’s down to jurisdictions to implement what’s appropriate within their jurisdiction” (P3c)

“So it’s been implemented in a voluntary way by each jurisdiction, so some jurisdictions are going to implement it and others probably won’t” (P10s)

Engagement with the food regulation system was an aim of the Food & Drink Reform project.

#### Food regulation

Study participants noted the incongruency between the public health and best-practice regulatory priorities in the updated FSANZ priorities, noting that ‘best practice regulation’ adds a significant barrier to achieving ‘chronic disease prevention’:

“Everything that food ministers try to do is checked by the Office of Best Practice Regulation, which says you have to do a regulatory statement that meets their standards… I think there’s something about the standard-by-standard assessment process that we have, that fails in terms of actually driving our food supply in a positive direction” (P6s)

Several policy areas were being considered at the time of data collection, including a review of the way ‘added sugars’ appear on food labels. A Forum Communique noted “the option to quantify added sugars in the nutrition information panel best met the desired outcome” and a “pictorial approach applied to sugary beverages… warrants further investigation” [51], omitting many recommendations from the review of labelling laws and policy [52]. For example, Recommendation 12, to group all ‘added sugars’ (as well as ‘added fats’ and/or ‘added vegetable oils’) in the ingredient list was omitted, a policy implemented in Canada [53]. The Commonwealth is committed to limiting all forms of regulation, a position discussed further below.

In the absence of regulatory change in the food system, there are some voluntary activities underway, such as the voluntary interpretive front-of-pack food labelling (Health Star Rating). Jurisdictions have noted the potential of these types of interventions, but also their limitations:

“Whether you want to be critic or not, the Health Star Rating stuff is a step in the right direction. It’s got a long way to go, before it’s really effective, but that’s really a piece of work that is helpful, I think longer term” (P6s)

A participant noted that a key barrier to pursuing regulatory approaches is the tendency towards having “very diverse views” (P3c) between industry and public health. The Commonwealth appears very supportive of maintaining the presence of an industry voice at all stages, even in the development of voluntary actions to improve food offerings, e.g. Healthy Food Partnership. The Tasmanian submission to the Senate Inquiry highlighted the strengths and weaknesses of engaging with the food industry through the Healthy Food Partnership. That submission argued that while the food industry is well placed to inform the “implementation and success of healthy eating strategies” and should therefore be “a key stakeholder or partner in the development of some initiatives”, careful consideration should be given to the “risk of undue commercial influence on the development of policy and guidelines” [54].

In addition to foods and beverages within the general food supply there are other food regulation opportunities with potential to support obesity prevention efforts in early childhood, such as speciality foods and formulas targeted at young children. Infant (birth to 12 months) and follow-on formulas (6–12 months) are “tightly regulated within the Food Standards Code” (P3c), Standard 2.9.1 (under review at time of data collection). However, toddler formulas or toddler milks (from 12 months) are not specifically regulated even though the term ‘follow-on’ is used in promotion of these products. Foods for infants, such as commercial complementary foods (from around six months), have some nutrient standards (maximum sodium and minimum iron) and labelling requirements (more than 4% added sugar must state ‘sweetened’), Standard 2.9.2. However, there are no standards for commercial ‘toddler foods’ (aimed at 12–36 months), which some participants identified as a problem:

“Some of the issues are coming out are in that toddler food area where some products have been identified as not being particularly healthy… having high sugar content, those sorts of things. I think industry would expect the market to sort itself out in this area, i.e. parents won’t buy it. But that always assumes an underpinning knowledge of what’s in a food” (P3c)

Access to appropriate long-life infant and young child foods can also be a problem in rural and remote Australian communities. A participant noted that in remote stores in the Northern Territory it can be difficult to find commercially available toddler foods in appropriate textures with sufficient iron for their needs.

#### A new national strategy for obesity prevention

While states working through COAG could initiate new policy agendas over Commonwealth resistance, they recognized the problems of implementation without national leadership. Participants noted that the COAG-led National Obesity Strategy was a voluntary, unfunded project. Driven by the states, it had no authority to ensure that each jurisdiction would act within their sovereign spheres. Without funding, the capacity to act would be limited. Participants felt that “having money will be absolutely key to drive more activity” (P8s), this would require National Agreements or NPAs. Study informants noted that the National Obesity Strategy was being driven fundamentally by the states and territories and wanted national leadership:

“I think we just need some leadership at the national level and at the moment it feels a bit like the cart leading the horse and often there are lots of nice sort of platitudes and statements but it is very reliant on jurisdictions” (P4t)

Other participants noted a key benefit of the National Obesity Strategy being progressed through the COAG Health Council to overcome political cycles and shifting priorities,

“[The] real benefit is that it then doesn’t matter about who’s in power because it’s about a majority government response to it. So, you can then have changes in government, and it won’t necessarily impact the implementation across the country. So, there are some real benefits to that COAG Health Council process” (P3c)

Study participants identified that a National Obesity Strategy should clarify the roles and direct national and subnational responsibilities, rendering ownership for obesity prevention less opaque:

“We anticipate that we would clearly identify which elements the Commonwealth is directly responsible for and which elements the jurisdictions are, and similarly probably for the [National] Prevention Strategy because we need to be clear on who’s doing what and where the overlap is so that we can actually achieve that outcome” (P3c)

Further, its role will be to articulate key priorities and measures to prevent obesity, including a focus on the First 2000 Days (as articulated in [55]). This would mark a new era in childhood obesity prevention in Australia.

Jurisdictions were further interested in a commitment to the development of action plans for a nationally consistent approach, with flexibility to “respond to opportunities” (P6s) for locally relevant implementation and align with domestic “government commitments, policies and strategies” (P7t). Overall, study participants were keen to ensure the strategy considered environments as well as settings and families:

“I think we’d definitely want to see aspects there around environments and that that’s not lost… The risk is that it could become too emphasised on things that just focus on individuals” (P8s)

“[We expect] domains of action from public education to awareness raising to marketing legislation to fiscal policy to community developments to monitoring and surveillance… that would include tax and regulations… as well as specific sub-populations where there may be need for more intensive efforts or specific programs” (P2s)

Currently, Australia does not have any national social marketing campaigns promoting healthy eating and being active. While jurisdictions were interested in a national social marketing strategy “around core foods… and being active” (P6s), they would like to have input into the content and retain flexibility for appropriate materials and “culturally and geographically appropriate” [56] local messages:

“I think you’ve always got to allow a certain amount of stuff to be bottom up versus top down” (P6s)

Study participants were also interested in a national monitoring system. Of importance for jurisdictions was that information collected will be comprehensive, comparable across Australia, and policy-relevant:

“We need to be able to track trends in obesity over time. We’ve been really challenged by that in Australia… where we haven’t had surveys that have been done in the same way, on the same population. What we’re tracking over time has been very unclear” (P6s)

Some jurisdictions suggested the establishment of a permanent national monitoring and evaluation system (with consistent periodic data collection). Such a system, which included the monitoring of weight status, movement, and nutrition, was generally desirable among study participants, whether that was controlled nationally or the standardisation of locally collected data:

“I think that the monitoring and surveillance stuff should be kept at the national level and done nationally, rather than hand it out to the jurisdictions” (P6s)

“One of the things is probably around standardisation, and so we’re comparing the same data… and information to base those things [policy decisions] on” (P7t)

Some study participants noted the limitations of the Australian Institute of Health and Welfare (AIHW), the national health statistics agency, and its capacity to produce policy-relevant data:

“There’s just not much happening in the coordinated policy sense, at the national level… That’s not to suggest that AIHW and NHMRC [National Health and Medical Research Council] aren’t doing some amazing stuff, because they are and we’ve worked with them really closely, but it’s just not coming out in a policy sense” (P4t)

However, there was a recognition that the development of such a system would need to overcome some barriers between jurisdictions and across sectors:

“The problem is though, that all the jurisdictions have now developed their little CATI [Computer-Assisted Telephone Interview] surveys that they have had going for 20 years and to stop doing that would be very challenging, because it would disrupt their trends over time. There would be some argy bargy [*sic*] around trying to restructure that” (P6s)

“What’s a bit more challenging to do is get a full monitoring and evaluation system around that… So I guess that’s one aspect, is just the context and the culture of different agencies that you’re working with” (P8s)

Working with differences such as these between jurisdictions to achieve national policy harmonisation is at the heart of Australian intergovernmentalism and obesity prevention.

#### Intergovernmentalism and obesity prevention

Study participants noted ongoing public sector and health service reforms have impacted on the nature of the relationships within and between organisational structures. Formal mechanisms for collaboration have been “dropped” (P9t) since the end of the NPAPH and “informal networks” (P6s) have risen in their place. Senior officials recognise the value of maintaining engagement with other jurisdictions at multiple hierarchical points:

“There’s regular contact across the jurisdictions at both senior and I suppose operational officer level, and there’s quite a lot of sharing of information on new resources, updates to policies, issues that we’re encountering, and how they are or have been addressed by different jurisdictions, also sharing of research” (P2s)

There was recognition among study participants that current intergovernmental activities for obesity prevention were limited in the extent of reach and impact for early childhood, e.g. the Food & Drink Reform was aimed at school-aged children. They noted that the National Obesity Strategy would seek to do more to try to fill this gap.

Among study participants pursuing obesity prevention through COAG processes was viewed as a pragmatic way to increase participation across jurisdictions and maintain obesity as a priority even as government leadership changes over time:

“The real benefit to that is it gives you the ownership from a Commonwealth and a jurisdictional perspective… then it doesn’t matter about who’s in power because it’s about a majority government response to it. So, you can then have changes in government, and it won’t necessarily impact the implementation of this across the country” (P3c)

“[Having] COAG approved or recommended strategies does give states who don’t have something like the Premier’s Priority [a strategic state-level priority to reduce childhood obesity by 5%] in place a mandate or an imprimatur to action some of this work” (P5s)

The limitations of the siloed processes of COAG were identified. Study participants noted that the pursuit of broad environmental action areas (once agreed to by health ministers) would require engagement with other sectors, especially to implement, and rationalised such an approach as cost-effective:

“…people working together would be cost effective. But sometimes you can’t get things moving. Because you can actually make connections at your level and they all agree to it but it’s still got to be supported by the people above to drive it. So that’s where I’m coming from. I think it’s important and it’s a commitment. With COAG you’ll get the ministers agreeing but then maybe at COAG they might need to consider having it a bit broader than just the health minister. Maybe at the national government level they might need to think about that and then drive it through” (P7t)

“If through the National Obesity Strategy, we can get higher-level engagement across whole of government that would be amazing” (P6s)

A further identified limitation was the pace through which agreements have been reached through the COAG Health Council, e.g. a participant noted that:

“…the next National Health Agreement has been in development for a while” (P1s)

Study participants noted the limitations to what COAG was able to achieve and advocated for improved institutional support for intergovernmental work:

“Look, we talk to our jurisdictional colleagues a lot, and that’s great, but that there are limits to what we can do… collecting information and feeding it up is done in an inconsistent way. And it would be useful, particularly in smaller jurisdictions, I think to have those. There are some economies of scale that I think all jurisdictions could draw from” (P4t)

Subnational study participants saw value in more formal collaborative mechanisms which are both vertical and horizontal in their structure. Study participants reflected that without clear governance and leadership, there is no commitment to achieving or maintaining national consistency, expressing a need for a “process on-going for how we continually work” (P6s). Concurrently, study participants felt a degree of flexibility in the implementation of obesity prevention measures was warranted, and that such flexibility was a benefit of being a federation:

“But you know, we do operate in the Australian government system, which means that states and territories do have different rules about things, and it’s not just in obesity… I don’t know that you can ever completely erase that or whether you’d want to” (P1s)

“There is an argument and I think there’s some truth in it that, if you allow different jurisdictions to do some different things, then that’s a good thing, because you get the leapfrog effect in the same way that the tobacco legislation happened over many years. I don’t think that we should make it so tight, that everybody has to do exactly the same things” (P6s)

## Discussion

The findings of this study indicate that public officials in different jurisdictions calculate the strategic possibility and barriers and the degree of state agenda setting but note the limited chances of structural change in the absence of central leadership and funding. Since the funding for the NPAPH was stopped in 2014 there has been no national preventive health framework in Australia. In the absence of national leadership states and territories pursued action on obesity through the intergovernmental mechanisms available to them. This avenue was available because of the ongoing structure of COAG, its Councils, and working groups [23]. The end of the NPAPH (2014) coincided with the total removal of prevention from the national agenda. The National Childhood Obesity Prevention Project (2016) was an attempt by states to push it back on the agenda. Similarly, as the senate can allow alternative views to emerge because it is often not controlled by the ruling party, the Senate Inquiry (2018) was an example of a minor party attempting to bring obesity prevention back on to the national agenda.

Stalled action since the Senate Inquiry at the national level reflects limited Commonwealth political will. In the space of obesity prevention policy, the Commonwealth has been absent in pursuing fiscal and regulatory policies within their control. Despite efficiency being a key premise of neoliberalism, arguments for the cost-savings and cost-effectiveness of preventive health interventions [57] have been insufficient to persuade the current government to act. This suggests limitations of evidence-based policy making in the Australian health paradigm [17]. The interviews in this study highlighted that there is little political appetite to bring in national legislative measures. This is most clearly highlighted in the major party responses to the Senate Inquiry. In their Dissenting Reports, both the Government (Coalition) and the Opposition (Labor) opposed recommendations for a sugar-sweetened beverage tax, mandatory front-of-packet food labelling, and introducing regulatory measures on the marketing of discretionary choices to children on broadcast media [48], while the states supported these measures (in interviews and submissions to the inquiry [54, 56, 58, 59]). This indicated a closing of obesity prevention agenda setting at the national level and took the issue from one Commonwealth forum (the Senate) to an intergovernmental forum (COAG), led by the states. At the closing remarks of the National Obesity Summit (2019), the national Sports Minister (not Health Minister) indicated the Commonwealth position was that the states and territories–through COAG–should have ownership for the health of the nation, “I look forward to driving change through COAG to get all the states and territories working together to create a healthier nation” [60]. These positions shifted the problem out to the states, leaving policy gaps at the national level.

Despite these gaps, states and territories used soft power to ‘lead from below’ [25]. To overcome ineffective hierarchical modes of policy making, coordination within and between subnational governments through exercising ‘soft power’ can influence national agendas by building good will across party lines while still allowing jurisdictions to respond to their own ‘domestic’ politics [24, 25, 27]. The work of the Food & Drink Reform project was promoted by the study informants as an example of jurisdictions working together across partisan lines. The successes of the Food & Drink Reform were followed by the announcement of the National Obesity Strategy. In the Australian institutional framework that included COAG, obesity policy emerged in a sporadic and disconnected way. Study informants noted their frustrations with different systems in each jurisdiction and the watering down of language to get consensus, and each ‘going back’ to their own jurisdictions with piecemeal versions of the original concepts then implemented.

On 29 May 2020 Australian leaders agreed to the cessation of COAG, and an ongoing role for a new National Cabinet (with only First Ministers of each jurisdiction) created during the COVID-19 pandemic. The new National Federation Reform Council will meet once a year to focus on a “priority national federation issue” [61], the first meeting (December 2020) focused on national emergency management and mental health. Before its cessation, there had been long-term disinvestment in the strategic management of intergovernmental relations, and inadequate governance mechanisms to address the complex social problems of our time–such as chronic disease prevention [26]. The loss of COAG represents a widening structural gap to support national obesity policy in Australia. Study participants noted their frustrations with the machinations of government. Nevertheless, despite the COAG agenda being set by the Commonwealth, it had ‘its own momentum’ [24]. Issues could gain traction as jurisdictions have dedicated internal mechanisms for intergovernmental work, whose regular contact with their intergovernmental counterparts were a ‘standard feature’ of public service [23]. COAG mechanisms included senior departmental officials as the knowledge holders of how to implement policy, bureaucrats participated as trusted extensions of Ministers and relationship-building extended within and between jurisdictions [27].

A strength of federalism is the diversity of forums–its complexity provides openings for new policy agendas. To achieve success in obesity prevention, Australian policy makers at all levels of government need to work across policy domains with “continuous interaction across jurisdictions” [23] to ensure policy coherence. Such a capacity was not present within COAG, nor exists within the new National Cabinet. The ‘institutional frailty’ of COAG [24] was highlighted by how quickly it was terminated during the COVID-19 crises. The upcoming National Obesity Strategy and the National Prevention Strategy represent a potential opportunity to encourage institutional mechanisms for preventive health in this vacuum, including identifying jurisdictional responsibility and funding opportunities.

An identified need for an intergovernmental preventive health agency has strong support across a range of efforts to prevent chronic disease. The Senate Inquiry suggested a National Obesity Taskforce, to be embedded in the Commonwealth Health Department (supported by Opposition) [48]. The National Obesity Summit (see Fig 2 and S1 File) proceedings identified that a coordinated approach is essential and “systems need to be created to accelerate collaboration and coordination between parties” [60]. The COAG Health Council consultation paper for the National Obesity Strategy public consultation suggested an intergovernmental forum to sit under COAG [55]. Public feedback from this consultation suggests public support for such an agency, recommending it to be “led by the Commonwealth (because a national approach is needed), centrally coordinated at a state/territory level and implemented at a local level” [62]. Such an agency ought to limit Commonwealth power to control the institution and establish a permanent organisational structure with a dedicated workforce and undertake an integrated approach (‘cross portfolio’ and across all levels of government) to policy formation and implementation [22–24, 26]. It is timely that both the National Obesity Strategy (subnational led) and National Prevention Strategy (national led) are being drafted concurrently, and an opportunity exists to harmonise these strategies for a truly national obesity prevention framework and establish an enduring national preventive health agency.

Establishing a permanent intergovernmental preventive health agency (broader than obesity, but its prevention as a key pillar) is needed to ensure that these complex social and health problems are addressed in a coordinated way. However, it is unlikely there will be support from the Commonwealth to establish such an agency. While Labor supported the Senate Inquiry recommendation for a National Obesity Task Force, the Coalition did not, noting in their Dissenting Report that it was a “structural solution rather than a strategic one and it is unclear how adding another layer of bureaucracy will lead to better addressing obesity policy issues” [48]. The institutional frailty of COAG, underpinned by ideological differences and decision-making gridlock, weakened its effective power. As an outcome of the post-COAG intergovernmental review, the architecture of the new National Cabinet Councils is classic neoliberalism/New Public Management: designed to answer discrete questions, “task-orientated and time-limited” [42], with support from temporary expert advisory groups. Under the new framework, there are insufficient structural mechanisms to support and coordinate the required long-term strategies to prevent obesity. There are also concerns about who will be given a voice in expert advisory groups, given the current Commonwealth government’s preference for industry inclusion. The changes to Australian intergovernmentalism pushes obesity back onto health, as the portfolios who most directly deal with its consequences, and with limited formal institutional opportunity to cooperate between jurisdictions. The ‘straight-jacket of neoliberalism’ ensures a focus on immediate priorities of the government and inhibits public sector funding for the long-term good of society [19, 63]. Healthcare services make up the vast majority of health portfolios and they are “designed for illness”, not prevention [64], and as such are not designed to solve complex systems problems. The prevalent view of obesity prevention and the nature of healthcare both centre the solutions to obesity on the person (personal responsibility). A recent study suggests that applying the health services mindset to prevention can actually make obesity stigma worse, because it translates to a focus on individual change [64]. A national prevention agency, with clear institutional mechanisms and authority, is warranted in this new intergovernmental architecture. It ought to have both structural *and* strategic elements to encourage cooperation and coordination for implementation–vertically and horizontally–but with clear caveats to focus on systems-level approaches away from a focus on personal responsibility.

Participants identified the need to have structural support to coordinate implementation. Additionally, they identified a need for a national (or nationally consistent) system to monitor the progress of implementing strategies that are likely to be different in each jurisdiction. In the development of a national system for monitoring progress, a balance between the reporting of progress on common objectives [24], which is useful for making policy decisions, with the limitations faced by smaller jurisdictions is required. Historic attempts for nationally consistent measurements of policy outcomes highlight flaws of such systems set up only to monitor discreet policy options [23]. Therefore, a prevention-focused set of data should be sought which is both accessible (affordable) and useful (timely and representative) for jurisdictions.

### Policy areas specific to the First 2000 Days

The First 2000 Days is emerging as a key life stage for obesity prevention efforts. It was prominent in the recommendations of Senate Inquiry [48], the National Obesity Summit [60], and the National Obesity Strategy evidence check [65] and consultation paper [55]. The Commonwealth Health Department is in the early stages of developing their First 1000 Days policy space. This would mark a change in Australian obesity prevention policy [14] and signals that a policy window may be opening. Two key opportunities for national consistency in this potential new policy space exist. The first opportunity is focusing on a key setting for young children–the ECEC sector, as approximately 57% of Australian children (from birth to five years) usually attended care [66]. An initial project could extend the Food & Drink Reform out from school settings to include the ECEC sector, as this is already covered by the Education Ministers Meeting (formerly COAG Education Council). In the long term, consideration could be given to driving nationally consistent approaches (while still allowing jurisdictions to meet the needs of their communities) to the ECEC sector across Australian jurisdictions. A recent study has noted the difficulties in harmonising nutrition-focused practices across jurisdictions [67]. Currently, the national regulatory body maintains an advisory and reporting role on the ECEC sector (reporting to the Education Ministers Meeting) and instruction to jurisdictions on how to meet national standards is not permitted.

The second opportunity is the marketing of foods and beverages to infants and young children. Study informants noted the inadequacies of the MAIF Agreement in terms of reducing the impact of marketing of breastmilk substitutes but that its coverage is not as broad as the WHO Code, because it does not cover complementary foods. That an independent review of MAIF Complaints Handling Process [68] returned the responsibility for monitoring the agreement to the Commonwealth Health Department is an indication of part of the problem with MAIF. Concurrently informants noted that toddler ‘milks’ and foods targeted at young children are co-located with infant formulas and complementary foods in supermarkets and should be included in considerations for ensuring a healthy food supply for infants and young children.

A recent audit of products available in the Australian food environment by a consumer group found products being marketing for children under 12 months with added sugar content as high as 40% [69], which is incongruent with the Food Standards Code (Standard 2.9.2). Another audit by the same group found substantially high added sugar content in toddler foods and noted these are not covered by the Food Standards Code [70]. Options to address this are warranted given that Australian children are not consuming foods according to the Australian Dietary Guidelines in the early years [11, 12], and iron intake is suboptimal [71]. In a UK study, many parents reported that they felt commercial foods were both safer and nutritionally superior compared to homemade foods and they felt these foods were highly convenient [72]. There is emerging (although limited) peer-reviewed literature on the nutritional inappropriateness of these foods [73, 74].

Research has shown that some food marketing undermines optimal nutrition, and consensus in international health supports regulatory intervention [37, 75–78], as industry self-regulation has been shown not to work [79]. The Commonwealth Health Department commissioned an evidence check in 2018 to support the promotion of breastfeeding [80]. Its findings about the inadequacies of MAIF are repeated in the Enduring Breastfeeding Strategy [81]. Nevertheless, evidence-based policy is a political challenge as well as a technical process of translating research into real-world settings [16], involving interplay “between facts, norms and desired action”, where ‘evidence’ is contestable [17]. A recent study about party-based framing relating to the marketing of discretionary choices found that the Coalition (current Government) frames obesity as ‘personal responsibility’ and Labor (current Opposition) has no fixed opinion on the issue [34], perhaps reflecting the ideological tension within the Labor party.

A Commonwealth study informant felt that the underlying problem with inappropriate foods aimed at infants and young children is a gap in parents’ ‘underpinning knowledge’. The adjoining solution to this framing is education for parents. This oversimplifies the problem as one of ‘personal responsibility’ at the family level rather than acknowledging the role the food industry can play in improving child dietary intake. It also detaches the solutions from government action in the food regulatory space.

### Advancing prevention through the food regulation system

The origins of Australia’s food regulation system to establish a national food market (S1 File) have influenced the ongoing prioritisation of the food industry over preventive health in legislative considerations. For example, the Office of Best Practice Regulation requires a set of standards be met to reach a threshold to undertake a regulatory approach (FSANZ Priority 3). There is no equivalent threshold test to support chronic disease prevention (FSANZ Priority 2).

These new FSANZ priorities highlight an example of institutional ‘polarised pluralism’ with Coalition/Liberal national/some state governments on one side and Labor states on the other side, causing a ‘centre-fleeing haemorrhage’ [82], resulting in two contradictory institutional priorities being established. While Priority 3 has an institutional procedure in line with New Public Management (i.e. Office of Best Practice standards), Priority 2 has no equivalent procedure to support it. A national prevention agency could lead the development of a set of standards to support chronic disease prevention for FSANZ. By virtue of being a national agency it could also provide some subnational political cover from pressure by different parts of the food industry (e.g. food manufacturing, sugarcane farming, or national supermarket chains), felt differently in each jurisdiction. Industry pressure is likely to inform some of the delays in rolling out implementation of Food & Drink Reform projects (national menu labelling, removing promotion of discretionary choices from state-controlled settings). Additionally, the Forum is always chaired by the Commonwealth Health Minister, with considerable power to ensure only the ‘lowest common agreement’ is reached [27] or through continual deferent of decisions so that no action occurs, often coupled with prolonged ‘hold outs’ [83]. For example, the NHMRC-led definition of discretionary choices was due in 2020, but then delayed until the full dietary guidelines are reviewed. The interim guides of the Food & Drink Reform are awaiting this definition to be finalised. Such a definition could also serve as the basis for the regulation of broadcast and online media marketing.

The industry-led self-regulation model is prioritised over the use of legislative measures, an ideological feature of Australian national politics. Criticisms of the NPAPH have highlighted that while states/territories implemented programs, the national government failed to use policy levers available to them [33]. Reflecting on Australia’s national policies to impact on childhood obesity in the last decade, neither the current nor previous national governments implemented Australia-wide policies solely within their control, instead supporting a self-regulation model. However, the self-regulation model is constructed in a way that industry can meet the standards that they set, and therefore continue their practices, even though the agreements themselves are not effective. In this way it can then be argued that it is ‘not necessarily a market failure’, and even when a self-regulation model is shown to not be effective (e.g. the MAIF Agreement) the Commonwealth seeks to ‘partner’ with industry to ‘enhance’ the existing self-regulation model. Increasing rates of early childhood obesity can be seen as an outcome of a market failure [84, 85], similar to illnesses related to tobacco use are a market failure, and as such require a move away from self-regulation model and towards government intervention.

It is evident that food/beverage and advertising industries are aware of the traction gained towards the restriction of marketing internationally and Australian subnational efforts. While there remains a critical lack of action on digital/online platforms, advertisers have updated codes to broadcast media self-regulation (noted in Fig 2) and made announcements to self-restrict discretionary choices advertising within 150m of schools [86, 87]. This latter decision is in response to actions taken by the Australian Capital Territory and announcements by the Queensland government to limit discretionary choices advertising in settings such as government buildings and billboards, public transport vehicles, transport hubs, and street furniture within their control (Food & Drink Reform, program four). The Australian Capital Territory successfully implemented a policy to remove discretionary choices advertising from their public transport vehicles and they were also able to show no net loss of revenue [88]. Although, such a strategy may prove more difficult in states with significantly larger outdoor advertising portfolios like Victoria and New South Wales [86], or for those governments who had childhood obesity platforms but whose ideology is resistant to such measures, such as South Australia or New South Wales. These steps by industry are attempts to delay, or even prevent, additional Australian jurisdictions implementing these types of policies. Lessons from successful implementation of food system regulations such as a sugar-sweetened beverages tax in Mexico highlight the need for flexible framing in different contexts to overcome embedded ideological resistance to government intervention [89].

Despite a resistance at the national level to frame obesity prevention as anything beyond personal responsibility, the political context of commissioned research such as the evidence check [65] for the National Obesity Strategy consultation paper [55] reflects that the COAG Obesity Working Group were interested in framing obesity as a social *and* commercial problem and sought solutions framed with that lens [17]. Through the Food & Drink Reform states/territories were pursuing subnational policy harmonisation, agreeing to a set of nationally consistent protocols with enough flexibility to use different policy instruments and focus on different settings to achieve a shared goal [22, 24, 25]. The unevenness and diversity of Australia’s economic geography is expanding; undertaking this approach within the Australian federation creates “a laboratory for natural experiments in policy that enhance opportunities for cross-jurisdictional learning through comparison of different approaches to related problems” [24]. The reality of Australia’s federated system means that a national approach to obesity prevention will likely be implemented slightly differently in each jurisdiction. It is encouraging that despite the loss of COAG, the progression of the National Obesity Strategy has continued (the Secretariat position maintained by Queensland Health). Childhood obesity continues to draw attention from the media. That lens validates the authority of subnational leaders to take ownership of its resolution, while concurrently promoting the Commonwealth’s role [25]. As a subnational bipartisan endeavour, the National Obesity Strategy is an example of states seeking to ‘lead from below’ [25].

A draft National Obesity Prevention Strategy was released in October 2021 for public feedback [90]. Key strengths of the strategy draft are the identification of the social and commercial determinants of obesity, the first ambition focuses on environments, and the significance placed on prevention with its addition to the title of the strategy. However, the strategy lacks commitment by governments to act, funding allocation, and timelines for implementation. The ‘examples of action’ under the strategies listed use vague, non-committal language and there are no clear mechanisms for accountability or transparency noted. Findings from this research suggest several priority recommendations for action for the early years. These include:

- Sustained funding at Commonwealth and state/territory levels and an enduring national prevention agency (with clear monitoring and surveillance that provides policy-relevant data) to support intergovernmental efforts.
- Clear accountability by identifying who is responsible for enacting each action.
- Align the strategy to other national plans, such as the upcoming National Prevention Strategy and develop a National Food and Nutrition Strategy.
- Fully implement the WHO Code and adhere to WHA resolutions using a regulatory framework.
- Reduce the exposure of children to marketing of discretionary choices across all government domains, including those aimed at early childhood (i.e. government settings, government-controlled assets, out-of-home advertising, and print, broadcast and digital/online media).
- Leverage off existing Food Codes to protect a broader range of foods and drinks (i.e. composition) aimed at children the early years.
- Use economic tools to not only increase access and affordability of core foods, but also to decrease access and affordability of ultra-processed discretionary choices.

This study had a number of strengths and limitations. To our knowledge, this is the first publication to consider the role of intergovernmentalism in obesity prevention from the perspective of senior public servants in all Australian jurisdictions. These insights will be of use in other multi-level governance systems such as the European Union and federated countries such as the USA, Malaysia, Nigeria and Canada, but also in countries whose politicians have wholeheartedly embraced New Public Management (e.g. the Netherlands or United Kingdom). A limitation of this study is that only one interview per jurisdiction was undertaken. While the aim was to ensure the most appropriate senior officials were interviewed, and authors felt a saturation point was reached, it may have inadvertently limited the diversity of perspectives. This study considered the social-structural determinants of early childhood obesity through a food and nutrition lens. While the findings touched on multiple policy settings (e.g. food regulation and ECEC settings) it did not specifically investigate other important environmental determinants of obesity (e.g. transport or urban planning).

## Conclusions

The First 2000 Days is beginning to be recognised as a key life stage for obesity and chronic disease prevention. It is important that the impacts of the food system on the First 2000 Days are taken into consideration when forming policies which are directed at the whole of population. While the National Obesity Strategy consultation did identify areas to address social and commercial determinants, it is important that consideration of these elements in implementation is not lost in a paradigm that focuses on personal responsibility in their implementation [34]. Reliance on slow, soft, and sporadic political change is unlikely to be sufficient to address the increase in chronic disease related to overweight/obesity and poor nutrition. There are considerable opportunities to develop new forms of chronic disease prevention collaboration within and between Australian governments, especially as the National Prevention Strategy and the National Obesity Strategy are still being developed and while intergovernmental relations are facing a ‘reset’ now that COAG has been disbanded. The New Public Management philosophy severely restricts the institutional mechanisms available for effective obesity prevention in Australia. State and territory study participants advocated strongly that central to a national approach to obesity prevention is the development of a permanent national prevention agency, whose remit can extend past short term political cycles and have the capacity to coordinate this complicated policy space. Without such an agency, the complex problem of obesity/chronic disease prevention will only be addressed in a piecemeal way.

## Supporting information

S1 File Supporting information for introduction. (PDF)

S2 File Supporting information for methods. (PDF)
