# Supplementary material for: Intergovernmental policy opportunities for childhood obesity prevention in Australia: Perspectives from senior officials
Source: PLoS One. 2022 Apr 28;17(4):e0267701. doi: 10.1371/journal.pone.0267701 (PMC9049527; doi:10.1371/journal.pone.0267701)
Supplement: S1 File — (PDF) [file pone.0267701.s001.pdf]

S1 File. Introduction: Background

Additional information on Australian intergovernmental forums

Australian intergovernmental policy making rested with 11 COAG Councils. The two relevant for the early prevention of obesity in childhood include the COAG Health Council (CHC) and the COAG Education Council [1], see Fig 1 in main text. The COAG Health/Education Councils are comprised of health/education ministers from all jurisdictions, with several subordinate working groups underneath comprised of government executive and senior health/education bureaucrats. The Early Childhood Education and Care (ECEC) sector is a key setting for interventions aimed at children before they begin school, as approximately 57% of Australian children (from birth to five years) usually attended care [2]. The ECEC sector is regulated nationally through the National Quality Framework (NQF) by an independent authority whose board reports directly to the COAG Education Council (see Link). The NQF is monitored and promoted through state and territory education departments (except WA, communities department), whose assessors report on the extent ECEC services are meeting the NQF Standards.

The federalisation of food policy in Australia was driven by commercial concerns – the need for consistent regulation to encourage national (and then trans-Tasman) markets for food. Public health was a secondary concern. In 1991 the states referred their powers over food regulation to a new federal National Food Authority, unifying food standards nationally for the first time. In 1996 Food Standards Australia New Zealand (FSANZ) was formed, governed by a Council of ministers representing the Commonwealth government, each of the states and territories, and New Zealand [3]. All FSANZ decisions on standards must be approved through the Australia & New Zealand Ministerial Forum on Food Regulation (the Forum), which separated out from COAG in 2015. The Forum upholds the Food Standards Code which covers general standards (including requirements for labelling), food product standards (e.g. composition of infant formula) and safety standards. The Forum membership includes Australian national, state and territory, and New Zealand food ministers and their respective department representatives (senior departmental officials) in the Food Regulation Standing Committee (FRSC) (see Fig 1 in text). Ministers from all jurisdictions have veto powers in the Forum. Unlike the CHC, where the role of Chair is shared among the jurisdictions, the Forum is always Chaired by the Commonwealth minister (for a detailed timeline of the history of FSANZ, see Link). After FSANZ was moved out of the COAG institutional structure the Health and Food Collaboration was established to maintain a connection between FSANZ and the COAG Health Council.

Another voluntary action, the Healthy Food Partnership, was established to foster engagement between government, public health, and the food industry. It has three work areas: food reformulation, portion size and food service (Fig 1). While it is being led by the Commonwealth Health Department and has a jurisdictional liaison group, it is not an intergovernmental forum. The Healthy Food Partnership has not made any public communications since late 2018 (for more information about the Healthy Food Partnership, see Link).

Additional information on national obesity prevention activities 2016-2019

The National Childhood Obesity Prevention Project was announced at the COAG Health Council October 2016 meeting, in response to the end of the NPAPH. The primary body of work under this project was called *Reduce Children's Exposure to Unhealthy Food and Drink* (which we refer to as Food & Drink Reform in this study). Queensland, a resources-driven and therefore economically powerful jurisdiction [4,5], led the coordination of this project through the Obesity Working Group. Membership includes health department bureaucrats from all jurisdictions, see Fig 1. The Food & Drink Reform sought collective action to limit both availability and promotion of unhealthy food to children, focused on options available to jurisdictions, across five actions. Three of the actions were settings-based, healthy food and drink offerings and promotion in school, health, and sport and recreation settings. The CHC released joint statements with the COAG Education Council and Meeting of Sport and Recreation Ministers to harmonise nationally consistent guidelines across sectors in 2018.

The fourth Food & Drink Reform action was about food promotion (excluding broadcast/digital media); where foods can be promoted (within government-controlled settings) and a scheme to identify what foods can be promoted in those settings. An interim guide has been produced to ensure national consistency as jurisdictions implement food promotion policies in government-controlled settings. The identification scheme is linked to work to update the definition of 'discretionary choices', defined in the Australian Dietary Guidelines in 2013 [6], sitting with the final action of the Food & Drink Reform. The fifth program of the Food & Drink Reform project, led by the Commonwealth Health Department, was the use of the food regulation system to reduce unhealthy food and drink exposure to children. It focused on a guide for nationally consistent roll out of menu board labelling legislation and a review of the term 'discretionary choices' to support the fourth program.

In 2017 food ministers announced new FSANZ priorities. While maintaining commitment to food safety, the first additional priority was to support public health objectives for chronic disease prevention [7] and in 2018 another additional priority that the food regulatory system be responsive through 'best practice regulatory approaches' was added [8].

The Select Sente Inquiry into the Obesity Epidemic in Australia (Senate Inquiry) ran between May-December 2018, submissions were made by the Western Australian, Tasmanian, Northern Territory and Australian Capital Territory Governments as well as the Commonwealth Health Department. The Senate Inquiry was led by the Australian Greens Party, a minor left party.

The final report of the Senate Inquiry made 22 recommendations, eight of these related to establishing a funded National Obesity Taskforce with "representatives from all knowledge sectors" across all levels of government and sit within the Commonwealth Health Department [9]. Its recommended tasks included the development of a national obesity strategy and national childhood obesity strategy, national education campaigns, national physical activity strategy (but no recommendation for a national food and nutrition strategy), and funding for the development and implementation of evidence-based community prevention programs. [9]. It also recommended Australia's food system including guidelines review, food labelling, menu board labelling, reformulation, tax on sugar-sweetened beverages, and marketing of discretionary choices.

The recommendations of the Senate Inquiry final report reflected much of the state and territory government submissions to the inquiry. The inquiry chair, from a minor party, authored the final

report. The Dissenting Reports by the Government and Opposition made clear that many of these recommendations were not a priority for either major national party. Consequently, there has not been much traction on the recommendations of the Senate Inquiry final report. The lack of Commonwealth response to the Senate Inquiry, and following the goodwill developed through the Food & Drink Reform, drove further subnational action. The COAG Health Council Obesity Working Group announced in 2018 they would develop a National Obesity Strategy (see Fig. 1 in main text).

The National Obesity Strategy commenced with a National Obesity Summit in February 2019, co-hosted by the Queensland Health Minister and the Commonwealth Sports Minister (not the Health Minister). Senior health department officials from multiple jurisdictions presented at the Summit, with attendance from representatives from government, public health, academia, and industry. Recommendations of the National Obesity Summit included: recognising the critical period for intervention from pregnancy through early childhood; restrictions on food marketing as a key area across all jurisdictions; the development of a national physical activity plan; establishing a statutory body to invest in long-term strategies, such as a national food and nutrition policy [10].

At the time of data collection, a public consultation for the draft National Obesity Strategy was being developed (by the Obesity Working Group) as was a draft of the National Prevention Strategy (led by the Commonwealth Health Department).

References

1. Australian Institute of Health and Welfare (AIHW). 2018. Australia's health 2018: in brief. Cat. no. AUS 222. Canberra: AIHW.

2. Australian Bureau of Statistics. 2017. 4402.0 Childhood Education and Care, Table 1. Available from https://www.abs.gov.au/AUSSTATS/abs@.nsf/DetailsPage/4402.0June%202017?OpenDocument

3. Healy M, Brooke-Taylor S, Liehne P. 2003. Reform of food regulation in Australia and New Zealand. Food Control 14(6):357-365. https://doi.org/10.1016/S0956-7135(03)00043-4

4. Keddie JN and Smith RFI. 2009. Leading from below: How sub-national governments influence policy agendas. AJPA 68(1):67-82. https://doi.org/10.1111/j.1467-8500.2008.00610.x

5. Chordia S and Lynch A. 2019. Constitutional incongruence: Explaining the failure of the Council of the Australian Federation 43(3):339-367. https://doi.org/10.22145%2Fflr.43.3.1

6. National Health and Medical Research Council (NHMRC). 2013. Australian Dietary Guidelines. Canberra: NHMRC

7. Australia and New Zealand Ministerial Forum on Food Regulation. 2018. Communique 28 April 2017. Available from https://www1.health.gov.au/internet/fr/publishing.nsf/Content/ministerial-forum-communiques

8. Australia and New Zealand Ministerial Forum on Food Regulation. 2018. Communique 29 June 2018. Available from https://www1.health.gov.au/internet/fr/publishing.nsf/Content/ministerial-forum-communiques

9. The Australian Senate. 2018. Select Committee into the Obesity Epidemic in Australia: final report. Canberra: Senate Printing Unit.

10. Deloitte. 2019. National Obesity Summit: Summary of proceedings on 15 February 2019. Available from www.health.gov.au/obesity
