# Supplementary material for: Intergovernmental policy opportunities for childhood obesity prevention in Australia: Perspectives from senior officials
Source: PLoS One. 2022 Apr 28;17(4):e0267701. doi: 10.1371/journal.pone.0267701 (PMC9049527; doi:10.1371/journal.pone.0267701)
Supplement: S2 File — (PDF) [file pone.0267701.s002.pdf]

S2 File. Supporting information for methods

A recent systematic review sought to establish standardised protocols for reporting reproducibility in the field of qualitative research [1]. The review identified twelve transparency criteria for qualitative studies. It also identified that those twelve criteria were not equally applicable across different types of study design and identified three types of study reproducibility – exact, empirical, and conceptual [1]. Of the twelve criteria identified in total, seven were applicable for empirical studies such as this. These seven criteria were used to develop a reproducibility reporting protocol for this study and are described in the table below with details of where to find the relevant information in the submission.

Table: transparency criteria, descriptions of criteria being met, location of information in submission

Transparency criterion [1]	Description of criterion being met [1]	Information location
Criterion 1: kind of qualitative method	"The authors clearly identify the type of qualitative research approach they adopted" [1] (p.1299)	The first sentence of methods section identified this as a qualitative case study.
Criterion 6: documenting interactions with participants	"The authors describe how each interaction was documented and the associated content" [1] (p.1299)	Below in S2 File Part A.
Criterion 7: saturation point	"The authors describe the precise criteria used to conclude that they have reached theoretical saturation" [1] (p.1299)	*Participant selection and recruitment* in the methods section.
Criterion 8: unexpected opportunities, challenges and other events	"The authors describe any unexpected opportunities, challenges, and other events, how they were handled, and their impact on substantive conclusions" [1] (p.1299)	There were no unexpected opportunities, challenges or other events to report and so these types of events had no impact on the study.
Criterion 9: management of power imbalance	"The authors describe specific strategies used to address power imbalance with specific participants" [1] (p.1300)	Below in S2 File Part A.
Criterion 10: data coding and first-order codes	"The authors describe the first- order coding methodology and present the full code list" [1] (p.1300)	*Data collection and analysis* in methods section and S2 File Part B, under Analysis Framework.
Criterion 11: data analysis and second- or higher-order codes	"The authors describe the second-order coding methodology and present the full code list" [1] (p.1300)	*Data collection and analysis* in methods section and S2 File Part B, under Analysis Framework.

Part A) Additional information for *Participant selection and recruitment* section in Methods

The process for interacting with participants was articulated in the approved ethics submission and documented as follows:

Each participant was sent an email describing the study, why they were being invited, and provided the Participant Information Sheet (PIS) and Consent Form along with the contact details of EE and The University of Sydney ethics office. Up to three emails were sent before the next potential participant from the same jurisdiction was contacted. Date and time of email, and number of emails sent were noted in an Excel spreadsheet (stored on a password protected secure server). Once the participant agreed to participate emails or phone calls were used to arrange a time for the interviews. The date and type of interaction (email/phone), and any queries or issues and solutions

were noted in the spreadsheet. All participants sent their signed Consent Form prior to interviews. All interviews were undertaken over the phone, audio recorded. The date and duration of interview were noted in the spreadsheet along with any notes for follow-up. After the interviews were transcribed, and email was sent to each participant offering an opportunity to review the transcribed interview before analysis was undertaken, which also reiterated protocols of study withdrawal. These emails/calls were also noted in the spreadsheet.

This item relates to transparency criterion 6: documenting interactions with participants [1].

Management of perceived coercion or potential for power imbalance:

In order to minimise the potential for power imbalance the following clauses were included in the Participant Information Sheet:

> *Being in this study is completely voluntary and you do not have to take part. Your decision whether to participate will not affect your current or future relationship with the researchers or anyone else at the University of Sydney.*
>
> *During the interview, you are free to stop the interview at any time. Unless you say that you want us to keep them, any recordings will be erased and the information you have provided will not be included in the study results, up to the point that we have analysed and/ or published the results. You may also refuse to answer any questions that you do not wish to answer during the interview.*
>
> *If you decide to take part in the study and then change your mind later, you are free to withdraw at any time. There are no consequences for withdrawing from this study. You can withdraw by sending an email to researcher Emma Esdaile at [EMAIL ADDRESSS] with your full name and a short statement that you would like to withdraw from the study. Please let us know at the time when you withdraw what you would like us to do with the information we have collected about you up to that point (e.g. give permission to use data already provided so far or remove all your information from our study records)…*
>
> *Research involving humans in Australia is reviewed by an independent group of people called a Human Research Ethics Committee (HREC). The ethical aspects of this study have been approved by the HREC of the University of Sydney [Project Number: 2017/507]. As part of this process, we have agreed to carry out the study according to the National Statement on Ethical Conduct in Human Research (2007). This statement has been developed to protect people who agree to take part in research studies.*
>
> *If you feel you need any additional supportive counselling services please make contact with an appropriate counselling service, such as Lifeline Australia on 13 11 14.*
>
> *If you are concerned about the way this study is being conducted or you wish to make a complaint to someone independent from the study, please contact the university using the details outlined below. Please quote the study title and protocol number.*

This information was reiterated at the beginning of each interview and included in the follow-up email sent after the interview about the participants reviewing their interview transcripts.

This item relates to transparency criterion 9: management of power imbalance [1].

<u>Part B: Additional information for *Data collection and analysis* in Methods</u>

DOCUMENT ANALYSIS TO INFORM INTERVIEWS AND INTERPRET FINDINGS

Intergovernmental websites and identified reports, documents, and communiques

COAG Health Council www.coaghealthcouncil.gov.au

- Health Ministers Communiques (10 during the study period)
- Announcements (none were relevant to early childhood obesity prevention)
- Reports on Childhood Obesity: Food & Drink Reform project
    - National Interim Guide to Reduce Children's Exposure to Unhealthy Food and Drink Promotion (endorsed in 2018)
    - Joint statement by Health Ministers and the Meeting of Sport and Recreation Ministers 'healthy habits by keeping kids active and ahead of the game' (interim statement, endorsed in 2019)
    - Goals, principles and recommended nutritional standards for food and drink choices in public sector healthcare settings (the interim guide, which was endorsed in 2020)
    - Good Practice Guide endorsed by Education and Health Ministers (and joint statement)
- Australian National Breastfeeding Strategy: 2019 and beyond

COAG Education Council www.educationcouncil.edu.au

- Education Ministers Communiques (13 during the study period)
- Joint statements from Food & Drink Reform project (covered under COAG Health Council documents)
- Review of the Australian Children's Education & Care Quality Authority (independent regulator of the early childhood education and care sector)

Meeting of Sport and Recreation Ministers (MSRM) Australian Department of Health > Sport

- Sport and Recreation Ministers Communiques (4 during the study period)

Food Standards Australia New Zealand (FSANZ) www.foodstandards.gov.au

- Food Ministers Communiques (six during study period)
- Australian food regulation priorities 2017-2021
- Food Standards Code
- Labelling Review: Labelling Logic report (released in 2016, but published in 2011)
    - Recommendation 12 (added sugars, fats or vegetable oils)
    - Recommendation 13 (mandatory declaration of *trans*-fats)
    - Recommendation 20 (revision of Standard 1.2.7, health claims)

National websites

Commonwealth Department of Health

- National Obesity Summit Overweight and Obesity
    - Presentations at the National Obesity Summit (15 February 2019)
    - Summary of Proceedings
    - Summit Program
- National Obesity Strategy Consultation > National Obesity Strategy

    - Evidence Review by Sax Institute: Population level strategies to support healthy weight (2019)
    - Evidence Review by Sax Institute: Addressing the social and commercial determinants of healthy weight (2019)
    - NOS consultation paper (2019)
    - NOS Consultation Report (2020)
- Independent review of the Marketing in Australia of Infant Formula (MAIF) Complaints Handling Process Maternal and Infant Health > MAIF

National Health and Medical Research Council (NHMRC)

- Nutrition NHMRC > Nutrition
    - Review of the 2013 Australian Dietary Guidelines
    - Discretionary Food and Drinks Review
        - Discretionary Foods and Drinks Expert Working Group NHMRC > DFaD Working Group

Health Star Rating http://www.healthstarrating.gov.au (supported by FSANZ)

- Review of the progress of implementation after two years (27 June 2014 to 26 June 2016)

Australian Senate: Select Committee into the Obesity Epidemic in Australia Australian Parliament House > Senate Committee

- Submissions by Australian Capital Territory, Tasmania, Northern Territory, and Western Australia Governments, and Commonwealth Health Department
- Senate Inquiry final report

The Healthy Food Partnership website

- Working groups
- Communiques

Australian Association of National Advertisers (AANA) www.aana.com.au

- Children's Advertising Code
- Food & Beverage Advertising Code (2019)

Australian Food & Grocery Council (AFGC) www.afgc.org.au

- Two voluntary initiatives:
    - Responsible Children's Marketing Initiative (RCMI)
    - Quick Service Restaurant Initiative for Responsible Advertising and Marketing (QSRI)
- Note: RCMI & QSRI officially transferred over to AANA authority from 1 July 2020 (complaints handled by Ad Standards)

Framework for document analysis

	Political nature of (early childhood) obesity	Features of the political system	Institutional considerations	Actions for (early childhood) obesity
**Document 1**				
**Document 2**				
**…**				

<u>Semi-structured interview tool</u>

This study is interested in the ways jurisdictions interact in the obesity prevention space under Australia's federated system. It will focus on the period from when the National Partnership Agreement on Preventive Health (NPAPH) ended until now. As well as policies which relate to the general population, this study would also like to highlight key areas for obesity prevention in early childhood (from conception until children enter school).

1. Can you reflect on the NPAPH and tell me what worked/what could be improved?
   - **Prompt**: Can you tell me about the NPAPH governance mechanisms?
   - **Prompt**: did jurisdictions establish communication networks, share experiences or coordinate action?
   - **Prompt**: can you tell me about the performance indicators used?
2. What effect did the NPAPH have on broader environmental and structural considerations?
   - **Prompt**: What effect did it have on policies about broader environmental issues, e.g. food or physical activity environment?
   - **Prompt**: Were there permanent structural changes to intergovernmental relations?
3. Since the end of the NPAPH are you in contact with your counterparts in other jurisdictions? Do you collaborate on obesity prevention?
   - Formal mechanisms (**prompt**: e.g. CHC or the Forum; other working groups)
   - Informal mechanisms (**prompt**: relationships between colleagues/ position equivalents in health departments in other jurisdictions)
4. What lessons can be learned from the NPAPH for the newly agreed COAG-led obesity strategy?
   - **Prompt**: what HCI programs have continued since the NPAPH in [jurisdiction]?
5. From a health department perspective, what are the key priorities for [Jurisdiction] in a national obesity strategy?
   - **Prompt**: Leadership, autonomy, funding, COAG collaboration, supportive communication structures, clear guidelines about consistent engagement with the commercial sector +/- industry
6. Can you tell me about COAG Health Council projects happening in this space?
   - **Prompt**: CHC Food & Drink Reform
7. Are there any projects for obesity prevention in the food regulation system?
   - **Prompt**: are there any issues with food regulation in the early childhood space?
8. Are there any strategies that you think should be implemented by the Commonwealth government to address childhood obesity?
   - **Prompt**: Regulation, Guidelines, Fiscal, etc. For example: a SSB levy; taxes; marketing regulation (e.g. mandatory rules around marketing of breastmilk substitutes, complementary foods and advertising to children); special food status for foods intended specifically for young children, e.g. RTU products;
   - **Prompt**: national plans for nutrition, physical activity, obesity

Analysis Framework

First order coding: The four initial codes that were used to categorise data and facilitate analysis were based on previous research by Head [2]. These were the political nature of obesity (early childhood), features of the political systems, institutional considerations, actions for obesity (early childhood). To facilitate second-order coding and study interpretation, the coding was separated out into intergovernmental obesity prevention venues including COAG Health Council food and drink reform, and national obesity strategy, The Forum (food regulation), and other. Below are the extraction tables for the first order coding. This item relates to transparency criterion 10 [1].

Second order coding: Was undertaken in the form of pattern coding [3]. This item relates to transparency criterion 11 [1].

Extraction tables for first order coding

COAG Health Council – Food and Drink Reform

	Political nature of (early childhood) obesity	Features of the political system	Institutional considerations	Actions for (early childhood) obesity
**P1s**				
**…**				
**P10s**				

COAG Health Council – National Obesity Strategy

	Political nature of (early childhood) obesity	Features of the political system	Institutional considerations	Actions for (early childhood) obesity
**P1s**				
**…**				
**P10s**				

The Forum – food regulation

	Political nature of (early childhood) obesity	Features of the political system	Institutional considerations	Actions for (early childhood) obesity
**P1s**				
**…**				
**P10s**				

Other relevant national policy areas (e.g. other forums, or Commonwealth-only areas)

	Political nature of (early childhood) obesity	Features of the political system	Institutional considerations	Actions for (early childhood) obesity
**P1s**				
**…**				
**P10s**				

References

1. Aguinis H and Solarino A. Transparency and replicability in qualitative research: The case of interviews with elite informants, Strat. Mgmt. J. 2019;40:1291–1315. doi: 10.1002/smj.3015

2. Head BW. Three Lenses of Evidence-Based Policy. Aust J Public Admin. 2008;67(1):1-11. doi: 10.1111/j.1467-8500.2007.00564.x.

3. Saldaña J. The coding manual for qualitative researchers. 2nd ed. London: SAGE Publications Ltd.; 2013
